# Effect of Biannual Mass Azithromycin Distributions to Preschool-Aged Children on Trachoma Prevalence in Niger: A Cluster Randomized Clinical Trial

**DOI:** 10.1001/jamanetworkopen.2022.28244

**Published:** 2022-08-23

**Authors:** Ahmed M. Arzika, Dallas Mindo-Panusis, Amza Abdou, Boubacar Kadri, Beido Nassirou, Ramatou Maliki, Amer F. Alsoudi, Tianyi Zhang, Sun Y. Cotter, Elodie Lebas, Kieran S. O’Brien, E. Kelly Callahan, Robin L. Bailey, Sheila K. West, E. Brook Goodhew, Diana L. Martin, Benjamin F. Arnold, Travis C. Porco, Thomas M. Lietman, Jeremy D. Keenan

**Affiliations:** 1 The Carter Center, Niamey, Niger; 2 Centre de Recherche et Interventions en Santé Publique, Birni N’Gaoure, Niger; 3 Francis I. Proctor Foundation, University of California, San Francisco; 4 Programme Nationale de Santé Oculaire, Niamey, Niger; 5 The Carter Center, Atlanta, Georgia; 6 London School of Hygiene and Tropical Medicine, London, United Kingdom; 7 Dana Center for Preventive Ophthalmology, The Johns Hopkins University, Baltimore, Maryland; 8 Division of Parasitic Diseases and Malaria, Centers for Disease Control and Prevention, Atlanta, Georgia; 9 Department of Ophthalmology, University of California, San Francisco; 10 Department of Epidemiology and Biostatistics, University of California, San Francisco; 11 Institute for Global Health Sciences, University of California, San Francisco

## Abstract

**Importance:**

Because transmission of ocular strains of *Chlamydia trachomatis* is greatest among preschool-aged children, limiting azithromycin distributions to this age group may conserve resources and result in less antimicrobial resistance, which is a potential advantage in areas with hypoendemic trachoma and limited resources.

**Objective:**

To determine the efficacy of mass azithromycin distributions to preschool-aged children as a strategy for trachoma elimination in areas with hypoendemic disease.

**Design, Setting, and Participants:**

In this cluster randomized clinical trial performed from November 23, 2014, until July 31, 2017, thirty rural communities in Niger were randomized at a 1:1 ratio to biannual mass distributions of either azithromycin or placebo to children aged 1 to 59 months. Participants and study personnel were masked to treatment allocation. Data analyses for trachoma outcomes were performed from October 19, 2021, through June 10, 2022.

**Interventions:**

Every 6 months, a single dose of either oral azithromycin (20 mg/kg using height-based approximation for children who could stand or weight calculation for small children) or oral placebo was provided to all children aged 1 to 59 months.

**Main Outcomes and Measures:**

Trachoma was a prespecified outcome of the trial, assessed as the community-level prevalence of trachomatous inflammation–follicular and trachomatous inflammation–intense through masked grading of conjunctival photographs from a random sample of 40 children per community each year during the 2-year study period. A secondary outcome was the seroprevalence of antibodies to *C trachomatis* antigens.

**Results:**

At baseline, 4726 children in 30 communities were included; 1695 children were enrolled in 15 azithromycin communities and 3031 children were enrolled in 15 placebo communities (mean [SD] proportions of boys, 51.8% [4.7%] vs 52.0% [4.2%]; mean [SD] age, 30.8 [2.8] vs 30.6 [2.6] months). The mean coverage of study drug for the 4 treatments was 79% (95% CI, 75%-83%) in the azithromycin group and 82% (95% CI, 79%-85%) in the placebo group. The mean prevalence of trachomatous inflammation–follicular at baseline was 1.9% (95% CI, 0.5%-3.5%) in the azithromycin group and 0.9% (95% CI, 0-1.9%) in the placebo group. At 24 months, trachomatous inflammation–follicular prevalence was 0.2% (95% CI, 0-0.5%) in the azithromycin group and 0.8% (95% CI, 0.2%-1.6%) in the placebo group (incidence rate ratio adjusted for baseline: 0.18 [95% CI, 0.01-1.20]; permutation *P* = .07).

**Conclusions and Relevance:**

The findings of this trial do not show that biannual mass azithromycin distributions to preschool-aged children were more effective than placebo, although the underlying prevalence of trachoma was low. The sustained absence of trachoma even in the placebo group suggests that trachoma may have been eliminated as a public health problem in this part of Niger.

**Trial Registration:**

ClinicalTrials.gov Identifier: NCT02048007

## Key Points

**Question:**

Are mass azithromycin distributions effective in eliminating trachoma when distributed only to preschool-aged children?

**Findings:**

In this cluster randomized clinical trial of 4726 children in 30 communities in Niger, communities with hypoendemic trachoma in which azithromycin was distributed biannually to children aged 1 to 59 months had a slightly lower prevalence of trachomatous inflammation–follicular and trachomatous inflammation–intense relative to communities treated with biannual placebo, but the difference was not statistically significant.

**Meaning:**

These findings do not support the administration of targeted antibiotic treatments as an elimination strategy in areas with hypoendemic trachoma.

## Teaser

This cluster randomized clinical trial assesses the effectiveness of biannual mass azithromycin distributions to children aged 1 to 59 months in an area of Niger with hypoendemic trachoma.

## Introduction

The World Health Organization (WHO) recommends mass azithromycin distributions for districts with endemic trachoma. During mass drug administration, the entire community is treated, regardless of trachoma status. However, children may benefit the most from mass azithromycin distributions because they are the most likely to be infected with *Chlamydia trachomatis*, the causative organism of trachoma, and also the most likely to have the inflammatory manifestations of infection.^1,2,3^ Children may experience other benefits of mass azithromycin treatments, including reductions in diarrhea, respiratory infections, malaria, and even mortality.^4,5,6,7,8^ It is well known that antibiotic treatment of the entire community also has unintended negative consequences, with the most important likely being antimicrobial resistance.^9,10^ Thus, investigators have attempted to study the effectiveness of targeted treatments to children in an effort to maximize the positive consequences and limit the negative consequences of long-term mass antibiotic distributions.^11,12,13,14,15^

We report the results of a cluster randomized clinical trial that assessed the effectiveness of biannual mass azithromycin distributions to children aged 1 to 59 months. The present study was performed in concert with the Macrolides Oraux pour Réduire les Décés Avec un Oeil sur la Resistance (MORDOR) trial, a larger study that assessed the effect of mass azithromycin distribution on child mortality in 3 countries in sub-Saharan Africa.^4,5^ The Niger site of MORDOR was located in an area with hypoendemic trachoma and thus provided an opportunity to determine whether administering mass azithromycin treatments to children younger than 5 years might be a feasible path forward for areas with low levels of trachoma. Cluster randomization was performed because the intervention was inherently community based. We hypothesized that mass azithromycin distributed biannually to preschool-aged children would result in less trachoma after 2 years compared with mass treatment with placebo.

## Methods

### General Study Design

The MORDOR trial was a cluster randomized clinical trial set in Malawi, Niger, and Tanzania that assessed the effect of mass azithromycin distributions on childhood mortality.^16^ The MORDOR study was purposefully designed as a large, simple trial with minimal data collection. Separate smaller trials were conducted in concert with the main MORDOR trial in each country to assess potential health benefits of mass azithromycin beyond mortality. The present study assessed prespecified trachoma outcomes from the smaller trial in Niger, which was a parallel-group, placebo-controlled, cluster randomized clinical trial conducted in 30 communities from November 23, 2014, until July 31, 2017. Communities were randomly drawn from the same pool of communities as the larger MORDOR trial, and the trial intervention was identical. Communities were randomized to biannual mass distribution of either azithromycin or placebo to preschool-aged children. A random sample of children from each community was monitored annually for a 2-year period for numerous health outcomes. The primary outcomes of the sister trial included malaria, anthropometry, and macrolide resistance and are reported elsewhere.^17,18,19^ Clinical trachoma was a prespecified secondary outcome, defined according to the WHO’s simplified grading system as the presence of trachomatous inflammation–follicular (TF) or trachomatous inflammation–intense (TI).^20^ This study followed the Consolidated Standards of Reporting Trials (CONSORT) reporting guideline. The study protocol is available in Supplement 1. Ethical approval for this study was obtained from the Committee on Human Research at the University of California, San Francisco, and the Institutional Review Board of the Nigerien Ministry of Health. Oral informed consent was obtained from parents or caregivers before each treatment and monitoring visit (ie, both before and after randomization).

### Setting

The trial was conducted in the Boboye and Loga departments in rural Niger. The prevalence of TF among children aged 1 to 9 years was estimated to be 4% to 5% in surveys performed between 2009 and 2011.^21^ No mass azithromycin distributions had been administered during the previous 5 years, and no trachoma-related hygiene or sanitation interventions were being administered during the time of the trial. Likewise, no seasonal malaria chemoprophylaxis programs were operating during the study period. The study area was purposefully chosen in an area with hypoendemic trachoma because trachoma elimination requires a TF prevalence of less than 5% in children aged 1 to 9 years, but it is not clear how to best treat communities in which TF prevalence hovers around the WHO’s 5% elimination target. Current guidelines support continued mass azithromycin treatments; however, at such low levels of infection, trachoma may simply disappear on its own, even in the absence of specific interventions.^22^

### Eligibility

The unit of randomization was the *grappe*, which is the smallest government-defined cluster of households for a health unit. Grappes with a population ranging from 200 to 2000 on the most recent government census were eligible. Of the 646 eligible grappes in the study area, 1 was allocated to a separate study,^23^ 615 were enrolled in the main MORDOR trial,^5^ and 30 were randomly chosen for inclusion in the present study. Because they were randomly selected, the 30 communities in the present study should be representative of the underlying population. All children aged 1 to 59 months and weighing at least 3800 g were eligible for treatment. For monitoring, repeated cross-sectional random samples of 40 children per community were selected from the preceding study census, with separate random samples drawn at each visit.

### Study Visit Timing

A door-to-door census was performed at months 0, 12, and 24 to enumerate all children aged 1 to 59 months in the community. Monitoring examinations were performed after the census at months 0, 6, 12, and 24, although by design, conjunctival photography was not performed at month 6. Treatment was administered at a separate study visit after all study examinations had been completed except for month 18 treatment, which was administered during the census.

### Intervention

Each eligible child was offered a single, directly observed dose of study drug every 6 months. Treatment was offered to all children aged 1 to 59 months enumerated on the most recent census. Azithromycin was administered as an oral suspension at 20 mg/kg, calculated by weight for small children and estimated by height for children who could stand.^24^ The oral placebo suspension was identical in appearance and was administered in the same way. Participants were instructed to report adverse events to designated village representatives. A formal survey of adverse events taken for children aged 1 to 5 months is reported separately.^25^

### Outcomes

A random sample of approximately 40 children per community was assessed at annual monitoring visits (ie, months 0, 12, and 24), with a new random sample drawn from the preceding annual census at each monitoring visit. Repeated cross-sectional random samples provided a valid estimate of prevalence at each time point, minimizing bias due to loss to follow-up. Conjunctival photographs were taken of the everted right superior conjunctiva of each child using a Nexus 5 smartphone (Google) coupled to a 3-dimensional printed smartphone attachment that provides magnification and external illumination (Corneal CellScope; University of California, Berkeley).^26,27^ Images were subsequently assessed by masked graders for TF and TI according to the WHO simplified grading system (prespecified secondary outcomes of the trial, expressed as a community-level prevalence). Photograding training was conducted by an ophthalmologist with extensive experience diagnosing trachoma (J.D.K.). All graders were required to achieve sufficient agreement (ie, κ of 0.6) with the consensus grade from an expert panel on a set of 100 photographs. A first round of grading was performed by nonophthalmologists (D.M.-P., A.F.A., and T.Z.); all photographs graded as possible, probable, or definite TF or TI were subsequently reviewed by 2 masked ophthalmologists (T.M.L. and J.D.K.) for the final grade. Dried blood spots were also collected from each child at each monitoring visit and subsequently processed for antibodies to *C trachomatis* antigens (pgp3 and CT694) with a multiplex bead assay performed on the Bio-plex 200 instrument equipped with the Bio-Plex manager software, version 6.0 (Bio-Rad) using previously described methods.^28^ Seropositivity was defined using a panel of external standards.^29^

### Randomization

Grappes were randomized without restriction and at a 1:1 ratio to the azithromycin or placebo groups by the trial biostatistician (T.C.P.) using the statistical program R, version 3.1 (R Foundation for Statistical Computing). The randomization sequence was concealed by randomizing all communities at the same time after enrollment. The Nigerien study coordinator was responsible for enrolling the study grappes; treatment was offered to all children aged 1 to 59 months based on the annual door-to-door census.

### Masking

The azithromycin and placebo used for the study were both manufactured by Pfizer Inc, with identical appearance and packaging. Study bottles were labeled with 1 of 6 treatment letters, with 3 corresponding to azithromycin and the other 3 to placebo. All participants, field workers, and investigators were masked to treatment allocation. Trachoma photograders and laboratory staff were masked to treatment allocation and to the study visit and study grappe identifier, implemented by assigning a random 6-digit number to each set of photographs and dried blood spot.

### Statistical Analysis

Data were analyzed from October 19, 2021, through June 10, 2022, using R, version 4 (R Foundation for Statistical Computing). The community-level prevalence of TF at month 24 was compared between the 2 groups in a Poisson model adjusted for baseline community-level prevalence. The TI and antibody seroprevalence outcomes were assessed in similar models. The magnitude of effect was expressed as an incidence rate ratio in the azithromycin group relative to the placebo group. Statistical significance was determined by permutation testing (10 000 Monte Carlo replicates). Serology results from children younger than 12 months were excluded from analysis owing to the potential presence of maternal antibodies. Age-dependent serological antibody responses and approximate simultaneous 95% CIs were estimated using cubic splines.^30^ Sensitivity analyses performed with square root–transformed linear models provided similar results. All analyses were performed in an intention-to-treat fashion. No interim analyses were performed. Sample size considerations were based on the primary resistance outcomes of the trial.^18^ An analysis of covariance formula was used to approximate the statistical power for the present analysis, which found 80% power to detect a 4% difference in the mean prevalence in TF between treatment groups, assuming a mean community prevalence of TF in the placebo group of 5% with an SD of 5% and a correlation of 0.65 between the baseline and month 24 community-level prevalence values at a significance level of 2-sided α < .05.

## Results

**Figure 1.**
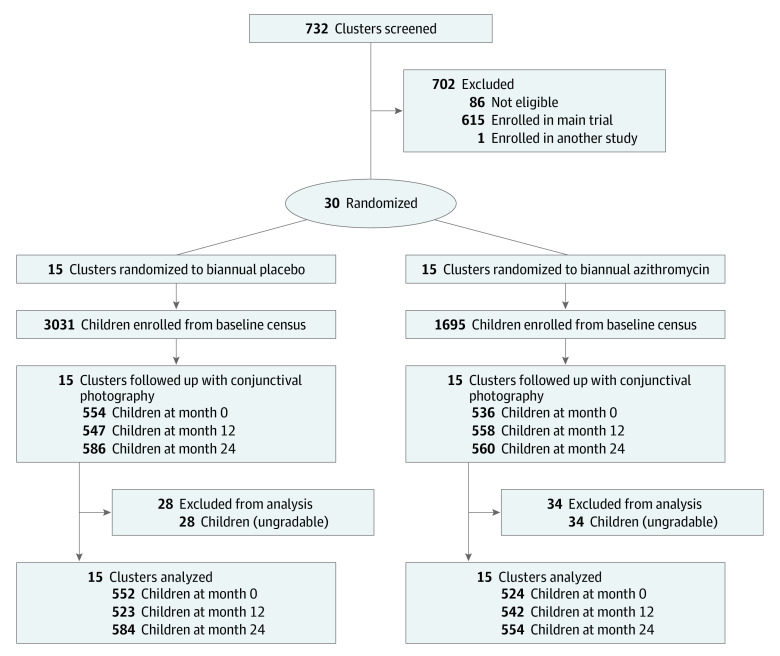
Study Flow Diagram All communities in the Loga and Boboye departments of Niger were screened for eligibility, with 86 excluded because the population was not in the desired range (ie, 200-2000 people). Of the remaining eligible clusters, 1 was allocated for an ancillary trial on antibiotic resistance,^23^ 615 were randomly chosen for the main child mortality trial,^5^ and 30 were randomly selected for the present trial. The 30 communities were randomized to biannual mass administration with either azithromycin or placebo for 2 years. Repeated cross-sectional random samples of children were selected from the preceding study census at months 0, 12, and 24 for conjunctival photography.

At baseline, 4726 children in 30 communities were included. Baseline characteristics were similar between 1695 children enrolled in 15 azithromycin communities (mean [SD] proportion of girls, 48.2% [4.7%]; mean [SD] proportion of boys 51.8% [4.7%]; mean [SD] age, 30.8 [2.8] months) and 3031 children enrolled in 15 placebo communities (mean [SD] proportion of girls, 48.0% [4.2%]; mean [SD] proportion of boys 52.0% [4.2%]; mean [SD] age, 30.6 [2.6] months), except that the mean (SD) number of children aged 1 to 59 months in the communities was smaller in the azithromycin group (113 [89] vs 202 [119] children) (Table).^10^ During the 4 biannual treatment visits, the study drug was administered to a mean of 79% (95% CI, 75%-83%) of children in the azithromycin group and 82% (95% CI, 79%-85%) of children in the placebo group (Figure 1). At the final month 24 monitoring visit, 554 children in the azithromycin group and 584 in the placebo group had photographs of acceptable quality and were included in the analysis (Figure 1). The 62 children with ungradable photographs were younger (mean [SD] age, 30 [17] months) than those with gradable photographs (mean [SD] age, 35 [17] months). No hospitalizations or life-threatening illnesses were reported in either group.

**Table.** Baseline Characteristics of the Study Communities Assessed From a Population Census^a^

Characteristic	Treatment group	
	Placebo communities (n = 15)	Azithromycin communities (n = 15)
No. of children aged 1-59 mo	202 (119)	113 (89)
Age distribution, % per community		
0 y	14.6 (4.8)	13.8 (4.6)
1 y	14.4 (3.9)	15.2 (5.1)
2 y	19.3 (3.2)	18.8 (5.0)
3 y	23.8 (4.4)	24.6 (5.2)
4 y	27.9 (6.9)	27.5 (5.8)
Sex, % per community		
Girls	48.0 (4.2)	48.2 (4.7)
Boys	52.0 (4.2)	51.8 (4.7)
Distance to nearest paved road, km	41.7 (27.1)	42.8 (18.9)
Distance to department capital, km	39.3 (22.7)	34.1 (18.8)
Elevation of community, m	203 (26)	210 (27)

^a^ All data are presented as mean (SD).

**Figure 2.**
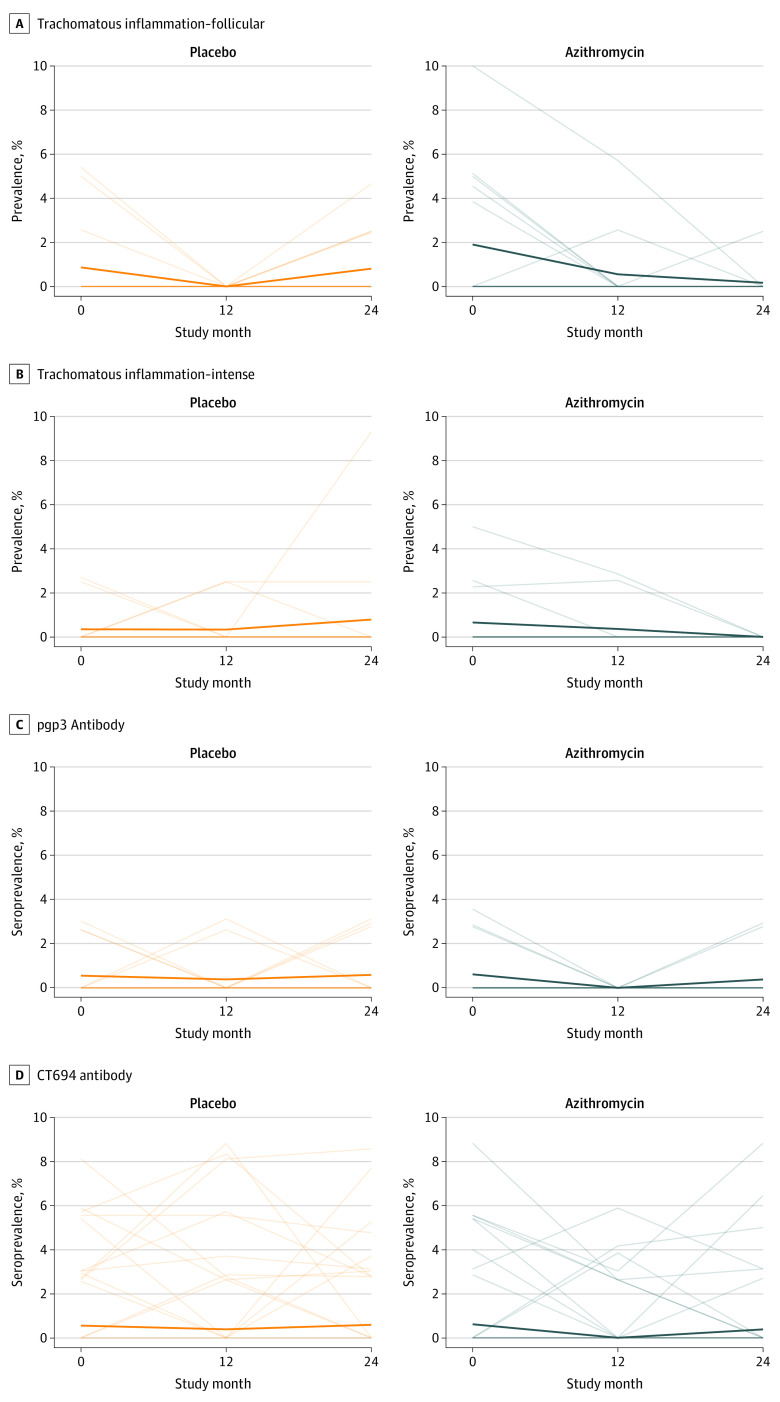
Longitudinal Prevalence of Trachoma Each thin line represents the prevalence of a study cluster over time, and the thick line represents the mean prevalence across all communities in the treatment group. A and B, Prevalence of trachomatous inflammation–follicular and trachomatous inflammation–intense among children aged 1 to 59 months; C and D, seroprevalence of pgp3 and CT694 antibodies among children aged 12 to 59 months.

The community-level prevalence of trachoma indicators over time is displayed for each group in Figure 2 and eTable 1 in Supplement 2. At baseline, the mean prevalence of TF was 1.9% (95% CI, 0.5%-3.5%) in the azithromycin group and 0.9% (95% CI, 0-1.9%) in the placebo group. Prevalence estimates clustered by community, with an intraclass correlation coefficient of 0.02. By month 24, the mean prevalence of TF was 0.2% (95% CI, 0-0.5%) in the azithromycin group and 0.8% (95% CI, 0.2%-1.6%) in the placebo group. The rate of TF was not significantly lower in the azithromycin-treated communities (incidence rate ratio adjusted for baseline, 0.18 [95% CI, 0.01-1.20]; permutation *P* = .07).

The mean prevalence of TI at baseline was 0.7% (95% CI, 0-1.5%) in the azithromycin group and 0.3% (95% CI, 0-0.9%) in the placebo group (Figure 2). The between-cluster intraclass correlation coefficient for TI at baseline was 0.002. The mean prevalence of TI at month 24 was 0 (95% CI, 0-0.7%) in the azithromycin group and 0.8% (95% CI, 0-2.2%) in the placebo group (permutation *P* = .96). Sensitivity analyses including both the month 12 and month 24 values produced similar results for both TF and TI. Results were similar in sensitivity analyses adjusted for population size (eTable 2 in Supplement 2).

**Figure 3.**
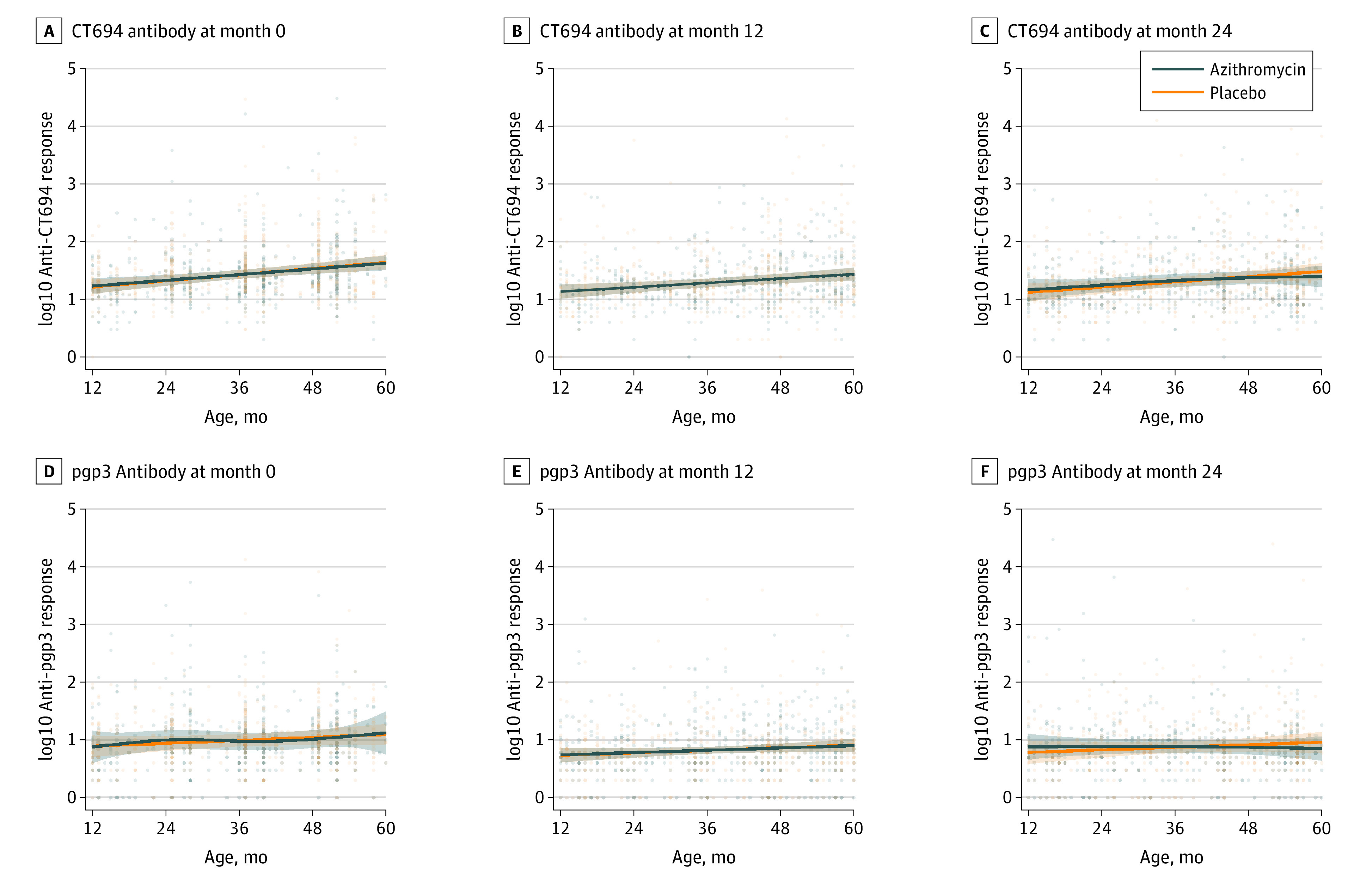
Chlamydia Antibody Responses Across Assessments Immunoglobulin G responses to CT694 and pgp3 are shown separately as smoothed curves of the continuous antibody response across ages, with the antibody response expressed as the log10 median fluorescence intensity minus background. Shaded bars represent approximate simultaneous CIs. Children in the azithromycin group are shown in blue and children in the placebo group are shown in orange.

Few children were classified as seropositive for chlamydia according to the external thresholds used for the study (eFigure in Supplement 2), and no child at any point had positive test results for both pgp3 and CT694 antibodies. Antibody responses did not display a marked increase with age, and antibody responses across ages did not differ noticeably between treatment groups across study visits (Figure 3). The mean pgp3 seroprevalence among children aged 12 to 59 months at baseline was 0.6% (95% CI, 0-1.3%) in the azithromycin group and 0.6% (95% CI, 0-1.1%) in the placebo group (eTable 3 in Supplement 2). At month 24, pgp3 seroprevalence was 0.4% (95% CI, 0-1.0%) in the azithromycin group and 0.6% (95% CI, 0-1.3%) in the placebo group (incidence rate ratio adjusted for baseline, 0.71 [95% CI, 0.09-4.30]; permutation *P* = .87). Analyses for CT694 seroprevalence were similar (eTable 4 in Supplement 2).

## Discussion

This study, set in an area of Niger thought to have hypoendemic trachoma, was designed to determine whether biannual mass azithromycin distributions to children aged 1 to 59 months would be sufficient for eliminating trachoma. This study question has public health relevance because targeting treatments to children could limit the spread of antibiotic resistance.^10^ In practice, both treatment groups had very low levels of trachoma even at baseline, and the difference between the groups was not statistically significant after 4 rounds of treatment.

The WHO has targeted trachoma for elimination as a public health problem, which it defines in part as a district prevalence of TF among children aged 1 to 9 years of less than 5%.^31^ Microbiological tests, such as serology or nucleic acid amplification tests, are not part of the definition of elimination. In this study, the prevalence of TF was well below the WHO elimination threshold of 5% at each study visit, albeit for children aged 1 to 59 months rather than 1 to 9 years. However, trachoma prevalence is usually higher among preschool-aged children than school-aged children, so it is likely that trachoma has been eliminated as a public health problem in this part of Niger.^32^

Serological tests for chlamydia were performed from dried blood spots for this study, which showed a lower chlamydia seroprevalence and flatter age-seroprevalence curves than other areas with more prevalent trachoma, consistent with low levels of chlamydia transmission.^33,34,35,36,37^ Indeed, no child in the study was seropositive for both pgp3 and CT694 antibodies, and titers from children who were seropositive were generally low, so even the few seropositive results may have been false-positive. Serological thresholds do not currently exist to differentiate areas with ongoing trachoma transmission vs those that have eliminated trachoma. Meta-analyses that include data from areas with varying levels of trachoma endemicity will be important to establish serological thresholds and standardize interpretation of serological data for trachoma.

Although not the subject of the present report, conjunctival swabs were collected from the random sample of 40 children at each monitoring visit during the trial. As reported elsewhere, a random set of 10 swabs per community was pooled and processed with metagenomic deep sequencing.^38^ Conjunctival swabs were found to harbor numerous bacteria, including *Haemophilus*, *Moraxella*, *Lactobacillus*, and *Streptococcus* species. Not a single *Chlamydia* species was discovered from the sequencing results at either the baseline or month 24 visit (ie, 0 prevalence across all 30 communities [binomial exact 95% CI, 0-1.2%]). Although processing the remaining swabs could tighten the 95% CI, ultimately these results are consistent with a lack of trachoma in the study area.

Few randomized trials of mass azithromycin distribution for trachoma have incorporated a placebo group. The finding that neither the clinical signs of trachoma nor the seroprevalence markedly increased in the placebo group during the trial, together with the consistent ocular chlamydia results, suggest that transmission of ocular chlamydia was low and that trachoma was unlikely to return even in the absence of any specific trachoma control activities.

This trial used a smartphone camera coupled to an external attachment to capture conjunctival images for clinical outcomes. This smartphone attachment, the Corneal CellScope, has been shown to be valid and reproducible for assessment of trachoma.^26,27,39^ As in previous reports, the quality of the smartphone images captured for this trial was good, with only 2% of photographs judged to be ungradable.^26,27^ Ungradable photographs were more likely to have been obtained for younger children, suggesting some association with participant cooperation. Nonetheless, conjunctival photographs are easy to mask, making them an appropriate choice as an outcome for a randomized trial. Although the lack of 3-dimensional data in a photograph theoretically could affect the diagnostic accuracy of a photograph, any misclassification would likely be nondifferential between groups and thus not bias the results.

### Strengths and Limitations

The strengths of this study include the placebo control group and the masked assessment of both clinical and serological trachoma outcomes. One limitation is that conjunctival swabs were not processed individually for chlamydial DNA, limiting the data available on the causative organism of trachoma. However, given the low prevalence of TF, TI, seropositivity, and chlamydia infections from pooled conjunctival swabs, it is unlikely that additional results from conjunctival swabs would change the overall conclusions. Another limitation was the relatively short 2-year study time frame. It is possible that a longer duration of treatment would have had an increased effect. The chief limitation was the extremely low prevalence of trachoma, which made it challenging to assess the efficacy of the intervention and limited its generalizability. It remains unclear whether azithromycin distributions to preschool-aged children would be effective in other areas with hypoendemic trachoma that have slightly more infection than those assessed in the present study.

## Conclusions

In this cluster randomized clinical trial, the low levels of trachoma in the study area made it difficult to determine whether azithromycin distributions targeted to preschool-aged children could be an effective elimination strategy for trachoma in areas with hypoendemic infection. The results of this 2-year, placebo-controlled trial suggest that trachoma has been eliminated as a public health problem in the Boboye and Loga departments of Niger and that ongoing trachoma interventions are unnecessary.

## Notes

Supplement 1.
Trial Protocol

Supplement 2. **eTable 1.** Trachoma Outcomes From Conjunctival Photography **eTable 2.** Analyses Adjusted for Cluster Population **eTable 3.** Community-Level Chlamydia Serology Outcomes **eTable 4.** CT694 Seroprevalence Outcomes **eFigure.** Distribution of Chlamydia Antibody Responses

Supplement 3.
Nonauthor Collaborators

Supplement 4.
Data Sharing Statement
