# Supplementary material for: Effect of Biannual Mass Azithromycin Distributions to Preschool-Aged Children on Trachoma Prevalence in Niger: A Cluster Randomized Clinical Trial
Source: JAMA Netw Open. 2022 Aug 23;5(8):e2228244. doi: 10.1001/jamanetworkopen.2022.28244 (PMC9399865; doi:10.1001/jamanetworkopen.2022.28244)
Supplement: Supplement 2. — eTable 1. Trachoma Outcomes From Conjunctival Photography eTable 2. Analyses Adjusted for Cluster Population eTable 3. Community-Level Chlamydia Serology Outcomes eTable 4. CT694 Seroprevalence Outcomes eFigure. Distribution of Chlamydia Antibody Responses [file jamanetwopen-e2228244-s002.pdf]

Supplementary Online Content

Arzika AM, Mindo-Panusis D, Abdou A, et al; Macrolides Oraux pour Réduire les Décés avec un Oeil sur la Resistance (MORDOR)–Niger Study Group. Effect of biannual mass azithromycin distributions to preschool-aged children on trachoma prevalence in Niger: a cluster randomized clinical trial. *JAMA Netw Open*. 2022;5(8):e2228244. doi:10.1001/jamanetworkopen.2022.28244

eTable 1. Trachoma Outcomes From Conjunctival Photography
eTable 2. Analyses Adjusted for Cluster Population
eTable 3. Community-Level Chlamydia Serology Outcomes
eTable 4. CT694 Seroprevalence Outcomes
eFigure. Distribution of Chlamydia Antibody Responses

This supplementary material has been provided by the authors to give readers additional information about their work.

© 2022 Arzika AM et al. *JAMA Network Open*.

eTable 1. Trachoma Outcomes From Conjunctival Photography

The community-level numbers of trachomatous inflammation—follicular (TF) and trachomatous inflammation—intense (TI) are shown for each community at each monitoring visit, as well as the total number of children with a gradable photograph.

	Month 0				Month 12				Month 24		
Grappe	TF	TI	Total		TF	TI	Total		TF	TI	Total
Placebo											
1	0	0	40		0	1	40		1	1	40
2	0	0	24		0	0	28		0	0	30
3	0	0	38		0	0	28		0	0	42
4	0	0	40		0	0	41		0	0	41
5	2	1	37		0	0	38		0	0	40
6	0	0	38		0	0	35		1	0	41
7	0	0	22		0	0	19		0	0	24
8	0	0	40		0	0	40		0	0	42
9	2	1	40		0	0	40		2	4	43
10	0	0	40		0	0	40		0	0	39
11	0	0	40		0	1	40		1	0	40
12	0	0	39		0	0	40		0	0	41
13	0	0	40		0	0	39		0	0	41
14	1	0	39		0	0	40		0	0	41
15	0	0	38		0	0	40		0	0	41
Azithromycin											
16	0	0	40		0	0	40		0	0	42
17	0	0	26		0	0	38		0	0	34
18	2	1	39		0	0	39		0	0	39
19	0	0	32		0	0	23		0	0	20
20	4	2	40		2	1	35		0	0	39
21	0	0	30		0	0	42		1	0	40
22	0	0	40		0	0	39		0	0	39
23	0	0	39		1	0	39		0	0	40
24	0	0	40		0	0	39		0	0	42
25	2	0	40		0	0	41		0	0	39
26	1	0	26		0	0	30		0	0	40
27	2	1	44		0	1	39		0	0	42
28	0	0	22		0	0	34		0	0	23
29	0	0	38		0	0	40		0	0	42
30	0	0	40		0	0	40		0	0	39

© 2022 Arzika AM et al. *JAMA Network Open*.

eTable 2. Analyses Adjusted for Cluster Population

Poisson regressions were performed comparing counts from the azithromycin and placebo arms at month 24, adjusted for baseline prevalence and baseline cluster population, and incorporating the number of children with test results as an offset.

Study visit	Incidence rate ratio (95%CI)	*P*-value
TF	0.21 (0.009-1.72)	0.21
TI	[a]	0.99
pgp3	1.22 (0.14–8.87)	0.84
CT694	0.66 (0.26–1.61)	0.37

[a] Invalid estimates in Poisson regression due to complete separation. A linear regression of community-level prevalence data was performed as a sensitivity analysis, which found the prevalence of TI was 0.7 percentage points lower in the azithromycin-treated communities (95%CI -2.1 to 0.7); *P*=0.33

© 2022 Arzika AM et al. *JAMA Network Open*.

eTable 3. Community-Level Chlamydia Serology Outcomes

The community-level numbers of pgp3 and CT694 seropositivity are shown for each community at each monitoring visit, as well as the total number of children with a dried blood spot.

	Month 0				Month 12				Month 24		
Grappe	pgp3	CT694	Total		pgp3	CT694	Total		pgp3	CT694	Total
Placebo											
1	0	3	37		0	1	36		1	0	34
2	0	0	23		0	0	26		0	1	27
3	0	1	33		0	1	27		1	1	32
4	0	1	39		0	0	38		0	0	34
5	1	0	33		0	1	35		1	1	36
6	0	1	34		1	0	32		0	3	39
7	0	1	18		0	1	18		0	1	21
8	0	2	35		0	3	36		0	1	36
9	0	2	34		1	1	38		0	0	36
10	0	1	37		0	3	34		0	0	38
11	0	2	37		0	0	37		0	2	38
12	0	0	34		0	1	38		0	1	33
13	1	0	38		0	0	35		0	0	35
14	1	1	38		0	3	37		0	3	35
15	0	1	33		0	2	35		0	1	36
Azithromycin											
16	1	2	36		0	1	38		0	0	35
17	0	0	20		0	0	30		0	0	25
18	0	1	32		0	2	34		0	1	32
19	1	0	28		0	1	24		0	1	20
20	0	2	36		0	1	33		1	3	34
21	0	0	29		0	0	39		0	0	32
22	0	0	35		0	0	39		0	2	31
23	0	2	37		0	1	38		0	1	32
24	0	0	40		0	0	38		1	0	36
25	1	1	35		0	0	36		0	0	35
26	0	1	25		0	0	14		0	0	35
27	0	0	39		0	0	34		0	1	37
28	0	0	30		0	1	26		0	0	20
29	0	3	34		0	1	38		0	0	37
30	0	2	37		0	0	37		0	0	36

© 2022 Arzika AM et al. *JAMA Network Open*.

eTable 4. CT694 Seroprevalence Outcomes

The mean CT694 seroprevalence across the 15 communities per arm is shown for the baseline and month 24 study visits.

	Mean prevalence (95% confidence interval)	
Study visit	Azithromycin	Placebo
Month 0	2.7% (1.3–4.2%)	3.2% (2.0–4.5%)
Month 24	1.9% (0.7–3.5%)	3.0% (1.7–4.4%)
Month 24 incidence rate ratio, adjusted for baseline: 0.64, 95%CI 0.27-1.43; permutation *P* = 0.33.		

© 2022 Arzika AM et al. *JAMA Network Open*.

eFigure. Distribution of Chlamydia Antibody Responses

Histograms depict the distribution of antibody responses to the pgp3 (panel A) and CT694 (panel B) chlamydia antigens. The red vertical lines represent seropositivity thresholds.

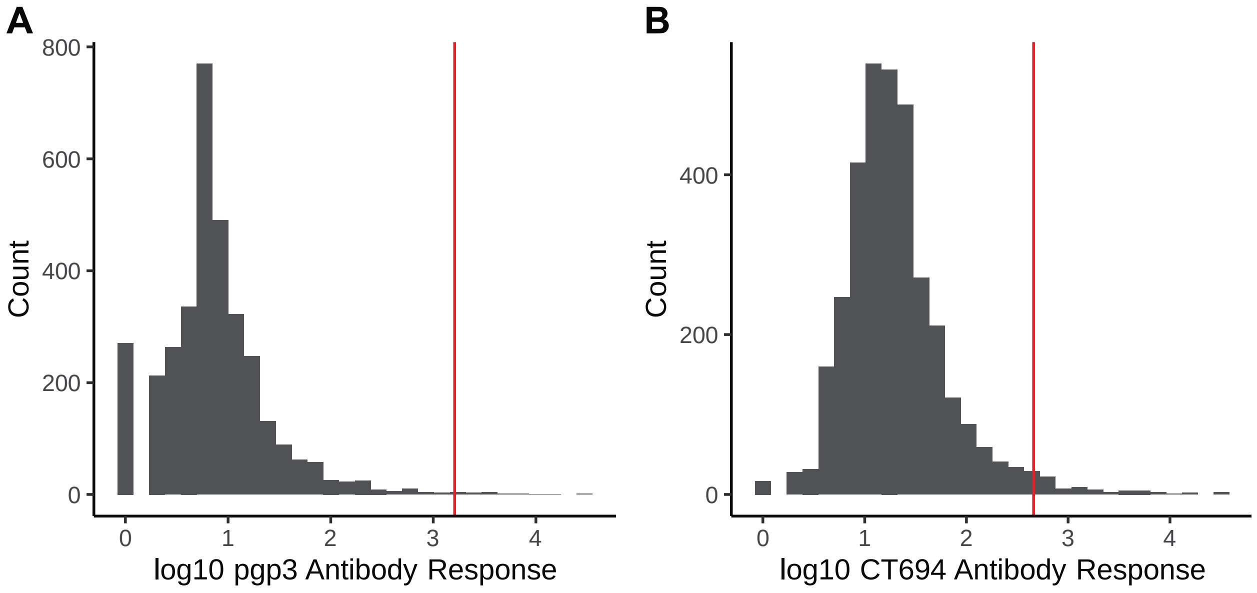

© 2022 Arzika AM et al. *JAMA Network Open*.
