# Supplementary material for: Effect of Biannual Mass Azithromycin Distributions to Preschool-Aged Children on Trachoma Prevalence in Niger: A Cluster Randomized Clinical Trial
Source: JAMA Netw Open. 2022 Aug 23;5(8):e2228244. doi: 10.1001/jamanetworkopen.2022.28244 (PMC9399865; doi:10.1001/jamanetworkopen.2022.28244)
Supplement: Supplement 3. — Nonauthor Collaborators [file jamanetwopen-e2228244-s003.pdf]

Supplemental Online Content: Nonauthor Collaborators
*First name, last name, and suffix (if applicable) are required and will appear in PubMed.

*Group Name(s): Macrolides Oraux pour Réduire les Décés Avec un Oeil sur la Resistance (MORDOR)–Niger Study Group							
***First Name and Middle Initial(s)**	***Last Name**	***Suffix (eg, Jr, III)**	Academic Degrees	Institution	Location (city, state/province, country)	Role or Contribution, eg, chair, principal investigator	Group (if more than 1 Group listed in the byline) and/or Subgroup (eg, Steering Committee)
Paul M	Emerson		PhD	International Trachoma Initiative	Decatur, GA	co-investigator	
Huub	Gelderblom		MD, PhD, MPH	International Trachoma Initiative	Decatur, GA	co-investigator	
PJ	Hooper		MA	International Trachoma Initiative	Decatur, GA	co-investigator	
Jerusha	Weaver		MPH	Johns Hopkins University	Baltimore, MD	co-investigator	
Sheila K	West		PhD	Johns Hopkins University	Baltimore, MD	co-investigator, steering committee	
Robin L	Bailey		MA, BM, M	London School of Hygiene and Tropic	London, UK	co-investigator, steering committee	
John	Hart		MD	London School of Hygiene and Tropic	London, UK	co-investigator	
Amza	Abdou		MD	Programme National de Santé Oculai	Niamey, Niger	co-investigator	
Nassirou	Beido		MS	Programme National de Santé Oculai	Niamey, Niger	co-investigator	
Boubacar	Kadri		MD	Programme National de Santé Oculai	Niamey, Niger	co-investigator	
Maria M	Ali		BA	The Carter Center	Niamey, Niger	co-investigator	
Mankara K	Alio		MD	The Carter Center	Niamey, Niger	co-investigator	
Ahmed	Arzika		MPH	The Carter Center	Niamey, Niger	co-investigator	
Nameywa	Boubacar		MD	The Carter Center	Niamey, Niger	co-investigator	
E Kelly	Callahan		MPH	The Carter Center	Atlanta, GA	co-investigator	
Sanoussi	Elh Adamou		MD	The Carter Center	Niamey, Niger	co-investigator	
Nana Fatima	Galo		RN	The Carter Center	Niamey, Niger	co-investigator	
Fatima	Ibrahim		RN	The Carter Center	Niamey, Niger	co-investigator	
Salissou	Kane		PhD	The Carter Center	Niamey, Niger	co-investigator	
Mariama	Kiemago		RN	The Carter Center	Niamey, Niger	co-investigator	
Ramatou	Maliki		MPH	The Carter Center	Niamey, Niger	co-investigator	
Aisha E	Stewart		MPH	The Carter Center	Atlanta, GA	co-investigator	

Supplemental Online Content: Nonauthor Collaborators
*First name, last name, and suffix (if applicable) are required and will appear in PubMed.

***First Name and Middle Initial(s)**	***Last Name**	***Suffix (eg, Jr, III)**	Academic Degrees	Institution	Location (city, state/province, country)	Role or Contribution, eg, chair, principal investigator	Group (if more than 1 Group listed in the byline) and/or Subgroup (eg, Steering Committee)
Cindi	Chen		MS	University of California, San Francisco	San Francisco, CA	co-investigator	
Catherine	Cook		MPH	University of California, San Francisco	San Francisco, CA	co-investigator	
Sun Y	Cotter		MPH	University of California, San Francisco	San Francisco, CA	co-investigator	
Thuy	Doan		MD, PhD	University of California, San Francisco	San Francisco, CA	co-investigator	
Bruce D	Gaynor		MD	University of California, San Francisco	San Francisco, CA	co-investigator	
Armin	Hinterwirth		PhD	University of California, San Francisco	San Francisco, CA	co-investigator	
Jeremy D	Keenan		MD, MPH	University of California, San Francisco	San Francisco, CA	co-investigator, steering committee	
Elodie	Lebas		RN	University of California, San Francisco	San Francisco, CA	co-investigator	
Thomas M	Lietman		MD	University of California, San Francisco	San Francisco, CA	principal investigator, steering committee	
Ying	Lin		MPH	University of California, San Francisco	San Francisco, CA	co-investigator	
Kieran S	O'Brien		PhD, MPH	University of California, San Francisco	San Francisco, CA	co-investigator	
Catherine E	Oldenburg		ScD, MPH	University of California, San Francisco	San Francisco, CA	co-investigator	
Travis C	Porco		PhD, MPH	University of California, San Francisco	San Francisco, CA	co-investigator, steering committee	

Supplemental Online Content: Nonauthor Collaborators
*First name, last name, and suffix (if applicable) are required and will appear in PubMed.

***First Name and Middle Initial(s)**	***Last Name**	***Suffix (eg, Jr, III)**	Academic Degrees	Institution	Location (city, state/province, country)	Role or Contribution, eg, chair, principal investigator	Group (if more than 1 Group listed in the byline) and/or Subgroup (eg, Steering Committee)
David A	Ramirez		MD	University of California, San Francisco	San Francisco, CA	co-investigator	
Kathryn J	Ray		PhD	University of California, San Francisco	San Francisco, CA	co-investigator	
Philip J	Rosenthal		MD	University of California, San Francisco	San Francisco, CA	co-investigator	
George W	Rutherford		MD	University of California, San Francisco	San Francisco, CA	co-investigator	
Benjamin	Vanderschelden		BSc	University of California, San Francisco	San Francisco, CA	co-investigator	
Nicole E	Varnado		MPH	University of California, San Francisco	San Francisco, CA	co-investigator	
John P	Whitcher		MD, MPH	University of California, San Francisco	San Francisco, CA	co-investigator	
Dionna M	Wittberg		MPH	University of California, San Francisco	San Francisco, CA	co-investigator	
Lee	Worden		PhD	University of California, San Francisco	San Francisco, CA	co-investigator	
Lina	Zhong		BS	University of California, San Francisco	San Francisco, CA	co-investigator	
Zhaoxia	Zhou		BS	University of California, San Francisco	San Francisco, CA	co-investigator	
