# Supplementary material for: Effect of Biannual Mass Azithromycin Distributions to Preschool-Aged Children on Trachoma Prevalence in Niger: A Cluster Randomized Clinical Trial
Source: JAMA Netw Open. 2022 Aug 23;5(8):e2228244. doi: 10.1001/jamanetworkopen.2022.28244 (PMC9399865; doi:10.1001/jamanetworkopen.2022.28244)
Supplement: Supplement 4. — Data Sharing Statement [file jamanetwopen-e2228244-s004.pdf]

Data Sharing Statement

Arzika. Effect of Biannual Mass Azithromycin Distributions to Preschool-Aged Children on Trachoma Prevalence in Niger. *JAMA Netw Open*. Published August 23, 2022. doi:10.1001/jamanetworkopen.2022.28244

Data
Data available: Yes
Data types: Deidentified participant data
How to access data: jeremy.keenan@ucsf.edu
When available: With publication

Supporting Documents
Document types: None

Additional Information
Who can access the data: Anyone requesting the data.
Types of analyses: For any purpose.
Mechanisms of data availability: Without investigator support.
